# A force measurement platform for a vitreoretinal surgical simulator using an artificial eye module integrated with a quartz crystal resonator

**DOI:** 10.1038/s41378-022-00417-8

**Published:** 2022-07-05

**Authors:** Yuta Taniguchi, Hirotaka Sugiura, Toshiro Yamanaka, Shiro Watanabe, Seiji Omata, Kanako Harada, Mamoru Mitsuishi, Tomoyasu Shiraya, Koichiro Sugimoto, Takashi Ueta, Kiyohito Totsuka, Fumiyuki Araki, Muneyuki Takao, Makoto Aihara, Fumihito Arai

**Affiliations:** 1 Department of Mechanical Engineering, The University of Tokyo, 7-3-1 Hongo, Bunkyo-ku, Tokyo, 113-8656 Japan; 2 Faculty of Advanced Science and Technology, Kumamoto University, 2-39-1 Kurokami, Chuo-ku, Kumamoto-shi, Kumamoto, 860-8555 Japan; 3 Center for Disease Biology and Integrative Medicine, The University of Tokyo, 7-3-1 Hongo, Bunkyo-ku, Tokyo, 113-0033 Japan; 4 Department of Ophthalmology, The University of Tokyo, 7-3-1 Hongo, Bunkyo-ku, Tokyo, 113-8655 Japan

**Keywords:** Engineering, Sensors

## Abstract

To provide quantitative feedback on surgical progress to ophthalmologists practicing inner limiting membrane (ILM) peeling, we developed an artificial eye module comprising a quartz crystal resonator (QCR) force sensor and a strain body that serves as a uniform force transmitter beneath a retinal model. Although a sufficiently large initial force must be loaded onto the QCR force sensor assembly to achieve stable contact with the strain body, the highly sensitive and wide dynamic-range property of this sensor enables the eye module to detect the slight forceps contact force. A parallel-plate strain body is used to achieve a uniform force sensitivity over the 4-mm-diameter ILM peeling region. Combining these two components allowed for a measurable force range of 0.22 mN to 29.6 N with a sensitivity error within −11.3 to 4.2% over the ILM peeling area. Using this eye module, we measured the applied force during a simulation involving artificial ILM peeling by an untrained individual and compensated for the long-term drift of the obtained force data using a newly developed algorithm. The compensated force data clearly captured the characteristics of several types of motion sequences observed from video recordings of the eye bottom using an ophthalmological microscope. As a result, we succeeded in extracting feature values that can be potentially related to trainee skill level, such as the mean and standard deviation of the pushing and peeling forces, corresponding, in the case of an untrained operator, to 122.6 ± 95.2 and 20.4 ± 13.2 mN, respectively.

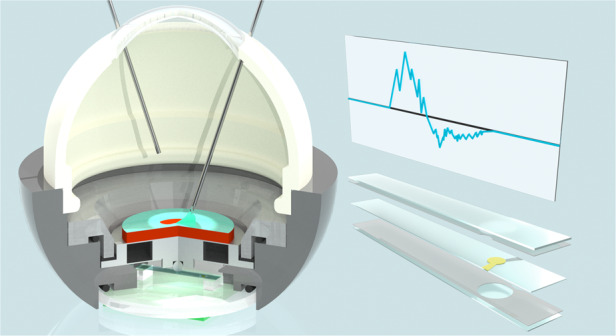

## Introduction

Ophthalmologists practicing intraocular surgery must acquire sophisticated skills to avoid injuring patients. To practice their skills, ophthalmologists generally perform surgical demonstrations using animal eyes; however, some of the structures of these do not resemble the structures of human eyes. Other training approaches involve mimicking the techniques of experts without the use of quantitative tactile sensation cues. Expert trainers also explain the skills needed for surgery in an empirical sense without quantitative indices. A further option is the use of virtual reality simulators^1^, most of which are expensive and do not provide a precise haptic sensation relating to the true substance of a human eye, which is important in improving surgical skill^2^. These training shortfalls mean that patients undergoing intraocular surgeries run relatively large risks of damage to their retina that depend on the skill of an ophthalmologist.

**Fig. 1:**
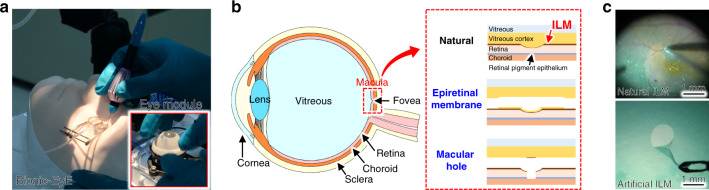
Training model for eye surgery and ILM peeling. **a** Image of the Bionic-EyE, the eye surgical simulator developed by the authors, with the eye module integrated with a sensor. **b** Structure of the inner eye and conditions occurring around the macular region (epiretinal membrane and macular hole). **c** Images of ILM peeling in an actual human eye and in an artificial ILM reproducing the mechanical properties of the eye^6^.

To address this issue, we attempted to improve upon common mock-up models^3–5^ by developing a “Bionic Eye surgery Evaluator (Bionic-EyE)”^6–8^ as a training model that can bionically reproduce the properties of human eyes with artificial materials. The Bionic-EyE is embedded with sensors for the evaluation of surgical skills and comprises an artificial eye module with disposable parts that enable trainees to perform repeated surgery simulations (Fig. 1a). Quantitative feedback from the sensor signals produced by the Bionic-EyE allows for the rapid acquisition of surgical skills. As a surgery that can be trained with the Bionic-EyE, we focused on inner limiting membrane (ILM) peeling, a complicated vitreoretinal surgery. As shown in Fig. 1b, the ILM is a thin and transparent membrane located between the vitreous cortex and retina^9^. The posterior part of the retina is called the macula and is responsible for a large part of the physiological visual field. The central part of the macula is called the fovea, which has a high density of cone photoreceptors and is responsible for photopic color vision with high acuity^10^. As humans age, the vitreous material in these regions liquifies and contracts, eventually leading to the detachment of the posterior vitreous cortex from the ILM of the retina in most individuals^11^. If the liquefication exceeds the degree of vitreoretinal dehiscence, a fragment of the vitreous can be left on the macula (epiretinal membrane), or a hole can be punctured through the fovea (macular hole) as a result of adhesive traction between the posterior vitreous cortex and the fovea^12^ (Fig. 1b). These effects lead directly to sight deterioration. Because the soft retina around the hole is pulled by the ILM, which is thick and stiff in elderly individuals^13^, the macular hole formed by this process does not close naturally. To treat this condition, the ILM is generally peeled off, as shown and reproduced by the Bionic-EyE^6^ in Fig. 1c. In some ILM peeling surgery procedures, increases in the closure of the macular hole and decreases in the recurrence rate have been reported^14–17^. The peeling of the ILM, which has a thickness of 3 µm^18^ and sticks to the retina, obviously requires well-matured skills in applying the appropriate force to the affected area. However, there is a lack of discussion in the literature on the use of force sensors in training models, although the use of forceps equipped with strain gauges^19,20^ or fiber Bragg grating force sensors^21–24^ has been attempted. For the practical demonstration of intraocular surgery, the skill needed to carry out ILM peeling should be quantified without adding sensors to the forceps. Our artificial eye module incorporates a quartz crystal resonator (QCR) force sensor and a strain body as a uniform force transmitter beneath a biomimetic ILM model, which is disposable to enable repeated use^7,8^.

A QCR force sensor is an oscillatory sensor with a self-excitation frequency that is linearly proportional to the force applied to its body^25,26^. Salient features of QCRs include their high-quality factors as oscillators, high mechanical strengths—particularly relative to flapping or torsional oscillators—and high carrier frequencies (>10 MHz). Based on these properties, we developed a QCR force sensor with high sensitivity and rigidity and a wide dynamic range^27–33^, allowing the QCR to detect a slight force even if a large initial force is applied in mounting the sensor within the eye module. These characteristics also reduce hysteresis and enable a rapid response to an applied force.

In this study, we used our QCR force sensor as the basis for developing a training platform for intraocular surgery through which the skill of ILM peeling could be quantified. The method for integrating the QCR sensor with an artificial retinal model was assessed in terms of the requirements for force sensitivity and uniformity over the entire ILM peeling surgical area. To enable practical use of the training module, drift compensation of the force signal was achieved using a novel algorithm, with which we evaluated forces during the peeling process. The skill in ILM peeling was characterized via compensated force signals obtained from the sensor and motion features captured by video during peeling.

## Results and discussion

### Design of the strain body

**Fig. 2:**
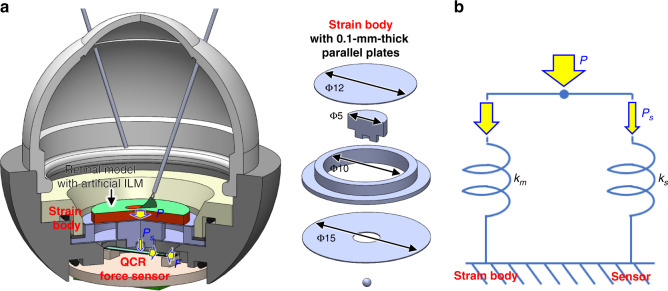
Design of the eye module with a force measurement structure **a** Conceptualization of the eye module comprising a beam-shaped QCR force sensor fixed at both ends and a strain body with 0.1-mm parallel plates. **b** Schematic static deformation model of a force measurement structure comprising parallel springs.

Figure 2a shows the design of an eye module with an integrated beam-shaped QCR force sensor and a strain body at its bottom. The strain body has a parallel-plate structure with a thickness of 0.1 mm, allowing it to avoid deformation in the torsional direction. The structure maintains a constant vertical displacement against any constant force applied to its upper surface regardless of the contact position within the ILM peeling area. In addition, the strain body is fabricated from stainless steel to ensure the exertion of preferred linear elasticity. The dimensions are expressed in Fig. 2a. The distance between the parallel plates is 2 mm.

Below the strain body, the beam-shaped QCR force sensor is fixed at both ends and contacts the hemispherical tip of the strain body. Part of an external force, *P*, applied to the strain body is propagated to the QCR force sensor as force $P_s$. In the sensor, $P_s$ is magnified to *F* and loaded onto the QCR domain of the sensing area. The force transmission efficiency from *P* to $P_s,\eta _1$ can be expressed by applying the static deformation theory of the parallel spring model (Fig. 2b) to the force measurement structure as follows:

1 $$\begin{array}{*{20}{c}} {\eta _1 = \frac{{P_s}}{P} = \frac{{k_s}}{{k_m \,+\, k_s}}} \end{array}$$

2 $$\begin{array}{*{20}{c}} {k_s = \frac{{192EI}}{{l^3}}} \end{array}$$

where $k_m$ and $k_s$ are the spring constants of the strain body and QCR force sensor, respectively, *E* is the elastic modulus of the quartz crystal in the chosen crystal orientation, and *I* and *l* are the area moment of inertia and the length of the beam-shaped QCR force sensor, respectively.

**Fig. 3:**
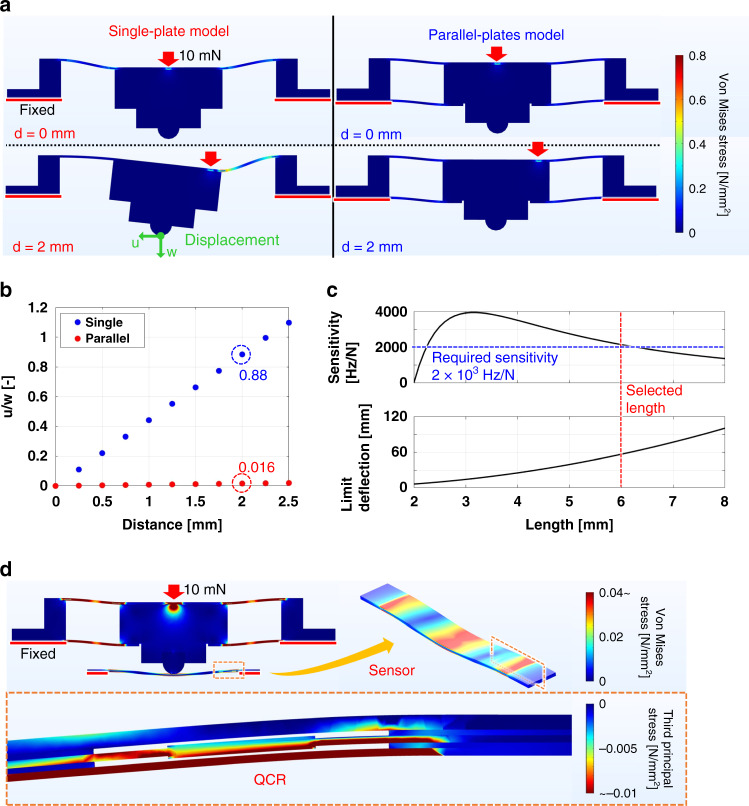
Finite element and theoretical analyses of eye module force measurement component performance. **a** Contour figure expressing distributions of Von Mises stress and deformation in single- and parallel-plate strain bodies with forces applied at 0 and 2 mm. **b** Plots of the ratio of horizontal to vertical displacement of the strain body tip at each position of applied force for single- and parallel-plate strain bodies. **c** Plots of the theoretical curves of model force sensitivity, $S_m$, and the maximum deflection of the sensor just before destruction, $\nu _{max}$, for different sensor lengths, *l*. **d** Contour figure of distribution of Von Mises stress on force measurement structure comprising parallel-plate strain body and beam-shaped QCR force sensor fixed at both ends, with figure illustrating the concentration of third principal stress in the QCR domain.

We analyzed the deformation of the strain body using a finite element model implemented in COMSOL Multiphysics (COMSOL, Inc.) and compared the results with those obtained for a strain body with a single plate on its upper side. The origin of the model was defined as the center of the strain body, and a constant force was applied to each point from 0 to 2.5 mm at intervals of 0.25 mm in the radial direction. Figure 3a shows contour plots for the single- and parallel-plate strain bodies under forces applied at 0 and 2 mm. At 0 mm, the stress is dispersed uniformly in both strain bodies; at 2 mm, however, the parallel-plate model disperses the stress, whereas the single-plate model experiences intense stress in one region of the plate. The spring constant of the parallel-plate model, $k_m$, was calculated to be 518 N/mm. The ratio *u*/*w* of the horizontal and vertical displacements of the strain body tip, *u* and *w*, respectively, was calculated for each point from 0 to 2.5 mm. Figure 3b shows that the ratio increases in proportion to the distance between the center and the point at which the force is applied in both the single- and parallel-plate strain bodies, with the parallel-plate model having a ratio approximately 55 times smaller than that of the single-plate model at each point. At 2 mm, the ratio of the single-plate model is 88%, which is close to 100%, whereas that of the parallel-plate is suppressed to within 2% (1.6%). These results confirm that the parallel-plate structure disperses the stress distribution uniformly by decreasing the horizontal displacement of the strain body tip and efficiently obtaining vertical displacement.

The dimensions of a QCR force sensor should be optimized to enhance its performance. We focused on the force sensitivity and maximal deflection of the sensor just before destruction. We first expressed $S_m$, the force sensitivity of the combined sensor and strain body, theoretically as the product of three parameters: $S_r$, the force sensitivity of the QCR; and $\eta _1$ and $\eta _2$, the force transmission efficiencies from *P* to $P_s$ and from $P_s$ to *F*, respectively ($\eta _2$ is defined in “Materials and methods”). $S_m$ must exceed the minimal value required for detecting the contact force of the forceps in ILM peeling, which is on the order of ~1 mN. We assumed that the force resolution should be below 0.5 mN and defined the minimal border as 2 × 10^3 ^Hz/N by dividing the noise fluctuation range of the sensor output, assumed to be 1 Hz, by the required force resolution. Subsequently, by approximating the QCR force sensor as a beam composed of a uniform quartz crystal and an isotropic linear elastic solid for simplicity of calculation, we calculated the degree of beam bending and obtained $\nu _{max}$, the maximal deflection of the sensor just before destruction, as

3 $$\begin{array}{*{20}{c}} {\nu _{max } = \frac{{\sigma _{max }l^2}}{{12Et}}} \end{array}$$

where $\sigma _{max}$ and *t* are the tensile strength of the quartz crystal and the thickness of the QCR force sensor, respectively. $\nu _{max}$ should be large enough to prevent the QCR force sensor from breaking when it is embedded within the eye module. During assembly, the sensor must be loaded with a sufficiently large initial force to achieve stable contact with the hemispheric tip of the strain body; a large maximal deflection also assists in the alignment of the sensor position. Therefore, it is optimal to obtain a $\nu _{max}$ that is as large as possible. The parameters of the force sensitivity $S_m$ and maximal deflection $\nu _{max}$ depend primarily on the sensor length *l*. A decrease in *l* improves $S_m$ but makes the assembly of the sensor more difficult because of the decrease in $\nu _{max}$; conversely, increasing the length makes the sensor more durable but less sensitive as $\nu _{max}$ increases and $S_m$ decreases. Thus, $S_m$ and $\nu _{max}$ have a trade-off relationship that should be optimized. Figure 3c shows the relationships between *l* and $S_m$ and $\nu _{max}$. As a value of *l* that can fit within the limited space at the bottom of the eye module while satisfying the requirements for both $S_m$ and $\nu _{max}$, we chose 6 mm. At this length, the theoretical sensitivity $S_m$ is 2.14 × 10^3 ^ Hz/N, corresponding to a force resolution of 0.47 mN.

We performed a finite element analysis of a force measurement structure comprising a strain body and QCR force sensor by applying an anisotropic material model to a quartz crystal to investigate whether the suggested deformation model is suitable from the viewpoint of structural mechanics. The results along the cross-section of the QCR force sensor shown in Fig. 3d illustrate that the QCR domain receives intense compression stress from the bending moment. Using the results of the analysis, the force sensitivity was calculated analytically from the compression stress applied to the QCR domain as 2.36 × 10^3 ^ Hz/N, a value close to the theoretical sensitivity.

### Performance of the fabricated device

**Fig. 4:**
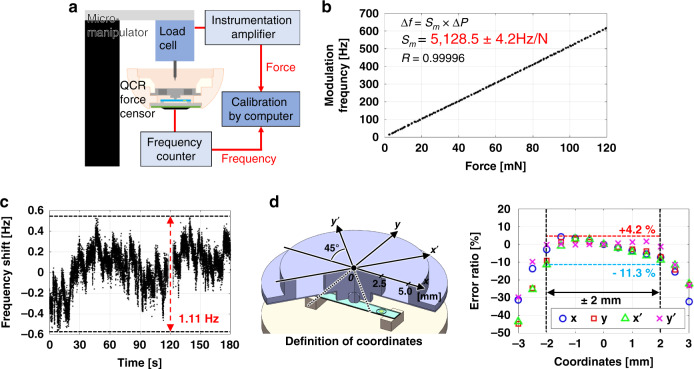
Evaluation of force measurement structure embedded in fabricated eye module. **a** Schematic of the system for calibrating force sensitivity. **b** Results of force sensitivity calibration. **c** Plots of frequency output fluctuation over 3 min. **d** Error ratios of calibrated sensitivities at multiple positions on the upper surface of the strain body relative to the sensitivity at the center, with the illustration of coordinate systems defined on the strain body for evaluation.

We initially calibrated the experimental force sensitivity using a load cell. Figure 4a shows the configuration of the calibration system. The calibration process revealed a fabricated force measurement structure sensitivity of 5.13 × 10^3 ^ Hz/N (Fig. 4b), which is 2.5 times larger than the theoretical and analytical values. It was estimated that the actual strain body deformed to a greater degree than had been analytically predicted because the adhesion between parallel plates was not perfect in the fabrication process, which in turn reduced the spring constant $k_m$. In addition, the output of the sensor as a function of applied force exhibited a high degree of linearity with a correlation coefficient of nearly one. Measurements of the noise level of the output of the QCR force sensor under static conditions for 3 min following driving and warming up for 2 h at a sampling rate of 100 Hz revealed a frequency output fluctuation range equivalent to 1.11 Hz, or 16 ppb (Fig. 4c). This equated to a force resolution of 0.22 mN, which appeared to be high enough for force detection during ILM peeling (for comparison, the force resolution of a fiber Bragg grating force sensor is 0.25 mN^21^). The maximum force measurable by the device was calculated to be 29.6 N, corresponding to a dynamic range of the eye module force measurement structure comparable to 1.3 × 10^5^. In addition, we confirmed that the QCR force sensor could respond to an input force within a sampling rate of 10 ms during calibration, proving that the rigidity of the sensor had little damping effect.

We subsequently evaluated the error in the force sensitivity in terms of the contact position on the strain body using the *xy*-coordinate system shown in Fig. 4d, in which the origin is located at the center of the strain body as defined on its upper surface. The *x* axis was directed along the length direction of the QCR force sensor toward the electrodes. An alternative *x'y'*-coordinate, aligned at an angle of 45° counterclockwise relative to the *xy*-coordinate system, was also defined, and we calibrated the force sensitivity at each point from −3 to 3 mm at intervals of 0.5 mm along each of the four coordinates and the error ratio for each obtained sensitivity relative to the value obtained at 0 mm was calculated. Within the expected 2-mm-radius ILM peeling area, the error ratio was suppressed to within a range of −11.3 to 4.2%. The beam-shaped QCR force sensor has a large positional dependence in terms of sensitivity, which leads to a loss in the reliability of measured force values; our results, however, indicate that the parallel-plate strain body has the effect of localizing the contact position with the sensor surface, thereby decreasing the error in the measured force value to a reliable level.

### Demonstration of ILM peeling

**Fig. 5:**
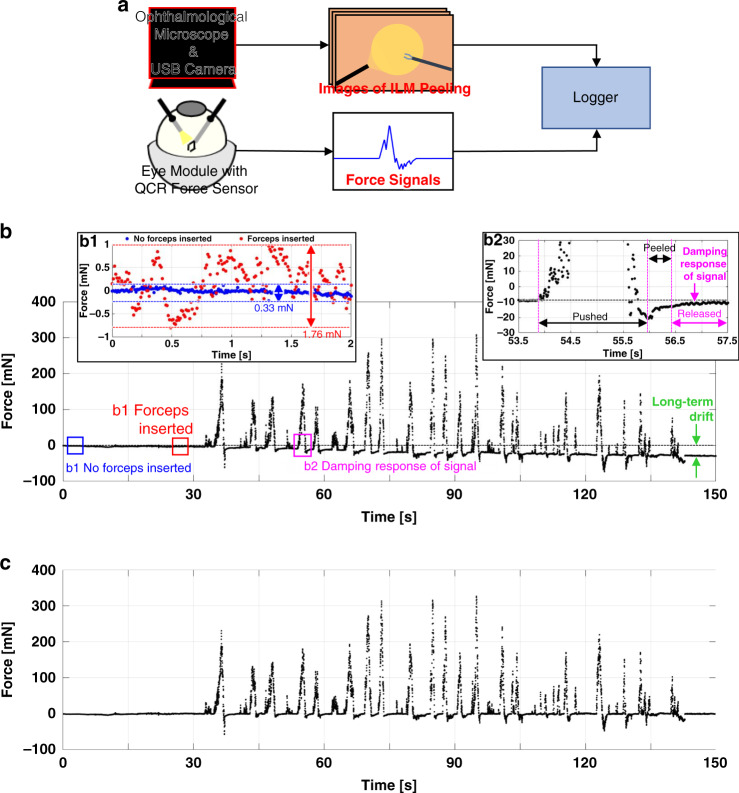
Demonstration of ILM peeling using the fabricated eye module. **a** Schematic of the evaluation system, which compares force signal produced by the eye module with video images obtained from the ophthalmological microscope. **b** Plots of the force signals produced in the ILM peeling process; **b1** plots of the partial data exhibiting fluctuation ranges of the force signals with and without forceps inserted; **b2** plot of the partial signal expressing damped response of the force signals just after forceps contact. **c** Plots of the compensated force signals throughout the ILM peeling process.

After embedding the fabricated eye module into the Bionic-EyE system, we measured the force values obtained from the beam-shaped QCR force sensor during the ILM peeling training. In the experiments, the eye module was fixed so that external disturbances arising from module motion would not affect the sensor signal. The ILM peeling simulation was performed using a participant with no specific experience or skills in carrying out the operation. During training, an ophthalmological microscope focusing on the bottom of the eye module recorded video (Fig. 5a).

Figure 5b shows the complete force signal produced over the ILM peeling exercise. In the ILM peeling simulation, two primary motions were generally observed: pushing by the forceps against the retina to grasp the ILM and peeling. The eye module outputs a positive force signal under pressing by the strain body and a negative signal under tractive motion, and a comparison between the force signal and video images confirmed that the detected positive and negative forces corresponded to pushing and peeling motions, respectively, throughout the simulation. In addition, the magnitudes of the pushing forces were in the range of a few dozen to hundreds of millinewtons, whereas the magnitudes of the peeling forces were on the order of dozens of millinewtons. The pushing-related force detected in the ILM peeling simulation might exceed the force measured in actual surgery corresponding to several to dozens of millinewtons^19,20^ because our retinal model has a higher elastic modulus (in the MPa range) than a real retina (20 kPa^34^). However, it was not crucial in this research because we did not evaluate the material properties of the retina but evaluated the index related to a surgical skill level in the simulation represented by the magnitude of the force.

Several experimental signals in addition to the signal produced by the retinal contact force during ILM peeling were also detected, representing, for example, the shaking of the forceps (Fig. 5b1) and the damped response of the force measurement structure (Fig. 5b2). In the initial state, in which the surgical instruments had not yet been inserted into the inner eye module, the eyeball contained only water, for which the signal fluctuation range was approximately 0.3 mN. Once the instruments had been inserted into the module, but prior to making contact with the strain body, the signal fluctuation increased to 2 mN. As a result, the shaking of the forceps propagated to the QCR force sensor via the eye shell as vibrations from the instrument insertion hole. However, the noise level of the shaking was smaller than the fluctuated signals of the contact of the forceps and therefore did not seem to be influential on distinguishing between the two. Signals that we believe were generated from viscous factors related to the force measurement structure were also detected, particularly immediately after the contact of the forceps with the retinal model (Fig. 5b2). This signal fluctuated within a range of a few dozens to hundreds of millinewtons during contact and then attenuated exponentially, possibly because the QCR force sensor and parallel plates of the strain body were fixed using resin materials.

### Drift compensation system

We also detected a long-term drift in the force signal throughout the ILM peeling process. The drift, which occurred at a rate of −20 mN every 2 min, might have arisen from factors such as the instability of the QCR oscillation owing to the high resonant frequency of ~70 MHz, electrical noise from stray capacitances produced by other components, or the temperature characteristics of the QCR. This long-term drift appeared to involve a gentle and linear change, allowing for compensation following signal recording.

**Fig. 6:**
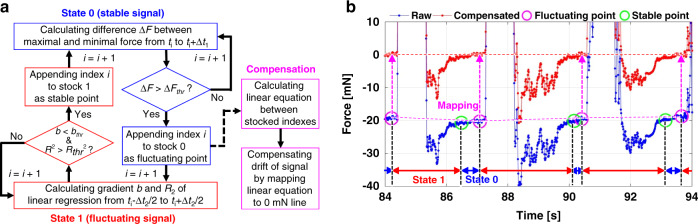
System for compensating the long-term drift of the ILM peeling force signal. **a** Schematic of the drift compensation system, which detects the instants of time just prior to the forceps contact and generates the offset signals based on these. **b** Results and details of the application of the compensation system on the force signal obtained in the ILM peeling demonstration.

In situations in which long-term drift occurs in the sensor signal, the contact force value during ILM peeling should be defined as the relative difference between the absolute value of the force on the signal-fluctuating region caused by forceps contact and the offset value of the force just prior to signal fluctuation. Therefore, we designed a system for automatically compensating the long-term drift of the force signal through the selection of an offsetting standard force value just prior to forceps contact. Figure 5c shows the compensated ILM peeling force data results. Figure 6a, b shows a schematic of the compensation system and the results of drift compensation, respectively. The procedure begins in the signal-stable state prior to contact, which is called state 0. In this state, the difference between the maximum and minimum forces, $\Delta F_i$, is calculated over the time interval from $t_i$ to $t_i + \Delta t_1$. Here, *i* expresses the number of time steps, and it is incremented in units of one until $\Delta F_i$ exceeds the force threshold, $F_{thr}$, with the corresponding $t_i$ set as the moment just prior to forceps contact and appended to state 0. The procedure then shifts into the signal-fluctuating state involving contact with the forceps, which is referred to state 1. In state 1, *i* is incremented, and the gradient *b* and coefficient of the definition $R^2$ of the linear regression of the force signal from $t_i - \Delta t_2/2$ to $t_i + \Delta t_2/2$ are calculated until the respective parameters simultaneously fall below and rise above the thresholds $b_{thr}$ and $R_{thr}^2$, respectively, at which point the signal is deemed to be stabilized by removal of the forceps from the retina and $t_i$ is appended to state 1 as the time at which the fluctuation of the signal stabilizes. The procedure then returns to state 0. In this manner, the times $t_i$ at which the signal fluctuation induced by forceps contact begins and the signal becomes stable are stocked by repeating this cycle until the end of the step. After detection, drift compensation is implemented based on state 0. At the beginning of the compensating process, the mean force value from $t_i - \Delta t_{offset}$ to $t_i$ is calculated as the offset value against each stock index *i* and used to derive a linear equation between neighboring detected points. Signal compensation can be carried out by mapping this equation to the 0-mN line against the raw force signal. Using this method, 36 of 40 contacts observed from the force signal and video information were accurately detected in our experiments.

### Evaluation of ILM peeling motions

**Fig. 7:**
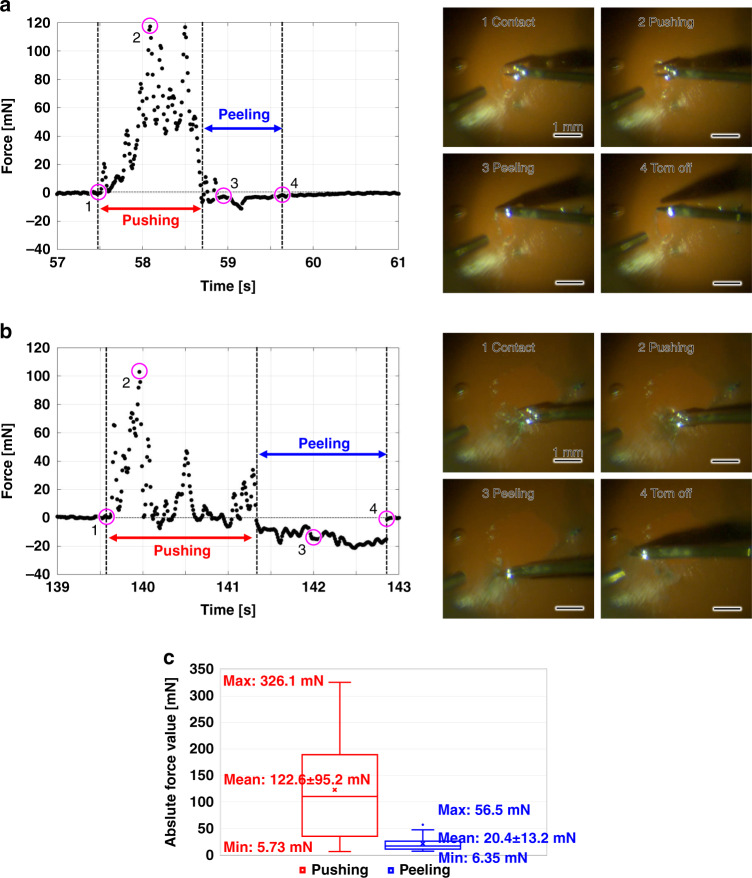
Characterization of the motion based on the ILM peeling force signal produced by an untrained individual. **a** Force signal generated by the ILM flap production. **b** Force signal generated by ILM peeling. **c** Distributions of the absolute force values around the means of pushing (*n* = 40) and peeling (*n* = 21) motions.

We evaluated the characteristics of the drift-compensated force data produced by the untrained participant. The ILM peeling action can be separated into two components: the task of cutting a flap in the ILM and the task of peeling the ILM by grasping the flap. Figure 7a, b shows a representative motion sequence for each component. In the former process (Fig. 7a and Supplementary Movie 1), a continuous positive force signal of ~120 mN is produced by pushing with the forceps to produce a flap, following which a short-lived negative force of approximately −10 mN is produced by transient tractive motion. In the peeling process (Fig. 7b and Supplementary Movie 2), there is a longer-lasting negative force of approximately −20 mN generated by peeling accompanied by intermittent positive force signals of ~100 mN prior to the peeling motion. In general, the pushing motion in the peeling component should be minimized to avoid additional damage to the retina, and our results indicate that the participant had room for improving the surgical skill.

As revealed by the force and video information, pushing motions were detected in all contacts produced throughout the demonstration process; peeling motions were detected in 21 of 40 contacts. Figure 7c shows box plots of the distributions of the absolute force values for the pushing and peeling motions, with means and standard deviations of 122.6 ± 95.2 and 20.4 ± 13.2 mN, respectively, indicating that the pushing force was larger and more widely distributed than the peeling force.

## Conclusions

In this study, we fabricated an eye module for ILM peeling training based on a force measurement structure comprising a beam-shaped QCR force sensor fixed at both ends and a stainless-steel strain body with a parallel-plate structure. The module was used to evaluate the contact force produced by forceps to a retinal model located at the bottom of the eye. Utilizing the QCR force sensor properties, including its high sensitivity and rigidity and wide dynamic range, the structure was able to achieve a force resolution of 0.22 mN, making it sufficiently sensitive to detect miniscule forces during ILM peeling, even under the initial loading on the order of several newtons applied by the assembly. The sensor had a highly linear output and responded rapidly to applied forces within a sampling rate of nearly 10 ms. The parallel-plate structure of the strain body served to suppress the force sensing error associated with the contact position on the strain body within a radius of 2 mm—the estimated radius of practical ILM peeling—to within a range of −11.3 to 4.2%. We demonstrated the measurement of the force in ILM peeling training and the compensating of the long-term drift of the QCR force sensor signal, which allowed us to correctly detect 36 of 40 contacts. Our results confirmed that contact forces ranging from dozens to hundreds of millinewtons were able to be distinguished using our eye module, enabling the extraction of some features of the ILM peeling motion produced by an untrained surgeon. In future work, we hope to use the eye module to log and analyze force information obtained from simulations conducted by ophthalmologists skilled in ILM peeling, which might potentially provide significant clues that will accelerate the improvement of the surgical skills of ILM peeling trainees.

## Materials and methods

### QCR force sensor

#### Mechanism

**Fig. 8:**
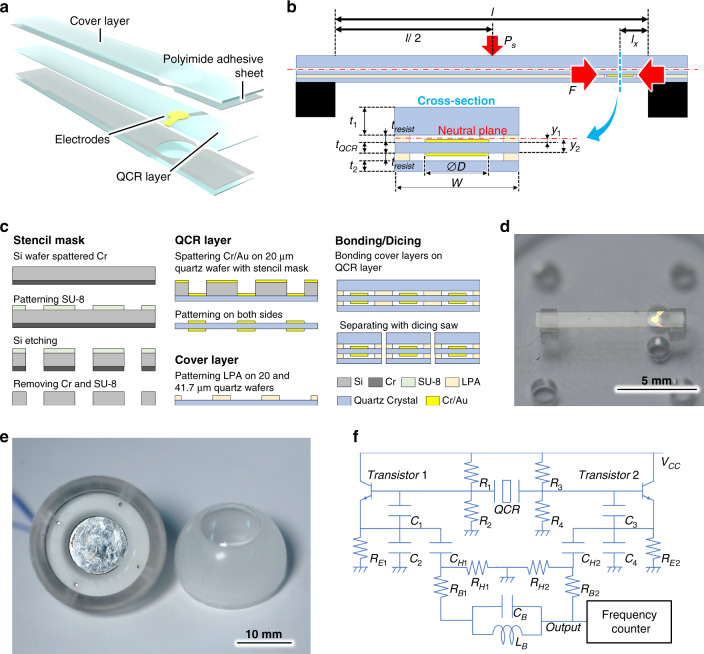
Design of the beam-shaped QCR force sensor. **a** Conceptual image of the beam-shaped QCR force sensor, comprising QCR-layer-patterned electrodes on each side, quartz cover layers, and polyimide adhesive sheets. **b** Illustration of the dimension and force measurement mechanism applied to the sensor. **c** Schematic of the sensor fabrication process, which proceeds from the application of a stencil mask to sputter the electrode pattern onto the QCR to depositing the QCR layer, patterning the cover layers using photosensitive adhesive, bonding, and, finally, dicing. **d** Image of the fabricated QCR force sensor. **e** Image of the fabricated eye module. **f** Schematic of the oscillation circuit.

The resonant frequency of a QCR shifts in proportion to the applied force as follows:^26^

4 $$\begin{array}{*{20}{c}} {\Delta f = S_r\Delta F} \end{array}$$

5 $$\begin{array}{*{20}{c}} {S_r = \frac{{K_ff^2}}{{nD}}} \end{array}$$

6 $$\begin{array}{*{20}{c}} {f = \frac{{1.67 \cdot n}}{{t_{QCR}}}} \end{array}$$

where *F*, *f*, $S_r$, $K_f$, *n*, *d*, and $t_{QCR}$ denote the force applied to the QCR, the resonant frequency, sensitivity, stress sensitivity coefficient, degree of overtone oscillation, electrode diameter, and wafer thickness of the QCR, respectively. The QCR force sensor comprises three layers, namely, a middle QCR wafer sandwiched between packaging quartz wafers, to increase its durability against force. To ensure a high Q-factor, the electrodes are kept in a vacuum environment^31^. To enable the sensing of forces on the micronewton order, we fabricated a beam-shaped QCR force sensor, as shown in Fig. 8a^32^. The position of the QCR, corresponding to the sensing domain, is displaced from the neutral plane of the beam-shaped sensor (Fig. 8b), allowing the QCR to receive an amplified force *F* as a result of the bending moment on the beam when the sensor applies a miniscule external force, $P_s$, to the upper surface. The efficiency of force transmission from $P_s$ to *F*, $\eta _2$, is expressed as:

7 $$\begin{array}{*{20}{c}} {\eta _2 = \frac{F}{{P_s}} = \frac{{MD}}{{2IP_s}}\left( {y_2^{\,\,2} - y_1^{\,\,2}} \right)} \end{array}$$

8 $$\begin{array}{*{20}{c}} {M = P_s\left( {\frac{1}{2}l_x - \frac{1}{8}l} \right)} \end{array}$$

where *M* and *I* are the bending moment applied to the QCR domain and the area moment of inertia of the sensor, respectively, *l* and $l_x$ are the length of the sensor and the distance between the electrodes and the nearest fixed end, respectively, and $y_1$ and $y_2$ are the distances from the neutral plane to the nearest and furthest surfaces of the QCR wafer, respectively. We decided to use a beam-shaped sensor based on the assumption that it would have sufficient force resolution to detect the force produced during ILM peeling, which is on the order of several millinewtons^19^.

To calculate the theoretical value of the model sensitivity, $S_m$, the relevant QCR force sensor parameters were set as follows: $l_x$ = 0.5 mm, thickness of thickest cover layer $t_1$ = 40 µm, thickness of the thinnest cover layer $t_2$ = 20 µm, thickness of the photoresist layer $t_{resist}$ = 15 µm, $t_{QCR}$ = 20 µm, *t* = 110 µm, $y_1$ = 0 µm, $y_2$ = 20 µm, *w* = 1 mm, *D* = 0.4 mm, *I* = $wt^3$/12, $K_f$ = 2.5 × 10^−11^ mm/(Hz‧N)^26^, *n* = 1, *E* = 72.5 GPa, and $\sigma _{max}$ = 150 MPa^35^.

#### QCR properties

An AT-cut quartz wafer was selected for the sensor in our research. It is generally known that the resonant frequency of an AT-cut quartz wafer is less dependent on a temperature change at ~25 °C^36^. Furthermore, we rotated the AT-cut wafer by 34.8° around the normal vector of the wafer plane to suppress the fluctuation of sensitivity due to temperature^35^. The diameter and thickness of the electrode were 0.4 mm and 250 nm of Au, respectively. The diameter size was defined by considering the width of the sensor beam and the area of adhesive between the QCR and the cover layers around the electrode. The beam width, 1 mm, was defined prior to the diameter to keep the maximum deflection and force sensitivity simultaneously within the limited space of the eye module. In addition, the diameter and thickness of the electrode were defined according to the theory of Bechmann^37^, which suggested that it should be required to balance the electrode diameter, thickness, and the decrease ratio of the resonant frequency due to the mass of the electrode (a decrease in frequency can be calculated from the Sauerbrey equation^38^). Following this theory, the resonant frequency was also determined to be ~70 MHz. The fabricated sensor oscillated at 69.396 MHz, around which it fluctuated in the range of 1.11 Hz every 3 min (Fig. 4c); thus, our design of the electrode is deemed to be appropriate.

#### Fabrication process

Figure 8c shows the process used to fabricate the QCR force sensor. First, a stencil mask for patterning the electrodes of the QCR was fabricated. A Si wafer, which had been sputtered with Cr film on one side, was rinsed with acetone for 10 min, followed by a denatured alcohol (Eta Cohol 7, Sankyo Chemical Co., Ltd) for 10 min, deionized water for 10 min, and, finally, piranha solution. The Si wafer was then spin-coated using a silane coupling agent of OAP at 1000 rpm for 10 s, baked at 145 °C for 30 min, spin-coated using SU-8 3050 (KAYAKU Advanced Materials, Inc.) as a negative photoresistor at 3000 rpm for 30 s and baked at 95 °C for 30 min, after which it was covered with a photomask patterned with the shape of the electrodes and exposed using an exposure machine (Suss MA6, SUSS MicroTech SE) at 40 mW for 7 s and finally baked at 65 °C for 4 min and at 45 °C for 1 min. The SU-8 pattern was developed using a PM thinner (Tokyo Ohka Kogyo Co., Ltd.) for 2 min, following which the wafer was baked at 150 °C for 4 min. Following deep reactive ion etching of the SU-8 patterned Si wafer with SPT MUC-21 ASE-Pegasus (Sumitomo Precision Products Co., Ltd.) and removal of the photoresists and Cr firm, the stencil mask process was completed. Subsequently, the QCR wafer was fabricated by placing a stencil mask on a 20-µm quartz wafer rinsed in the same manner as the Si wafer and spattering 10 nm of Cr and 250 nm of Au using a CFS-4EP-LL i-Miller (Shibaura Mechatronics Corporation) onto both sides. The cover layers were processed by exposing 20- and 41.7-µm quartz wafers laminated with negative photosensitive adhesive sheets (LPA, Toray Industries, Inc.) on each side for 22.5 s, following which wafers were developed using 2.38% TMAH (Tokyo Ohka Kogyo Co., Ltd.) for 90 s, left for 30 min, baked at 110 °C, and left again for 10 min. Finally, bonding cover layers were pressed onto both sides of the QCR wafer with a force of 500 N at 70 °C for 30 s, followed by baking at 200 °C for 1 h. The bonded wafers were cut with a dicing saw (DAD3650, DISCO Corporation) to separate them into each QCR force sensor. Both ends of the fabricated QCR force sensor were attached to the support stands on the acryl plate (Fig. 8d) using UV-curable adhesive (LOCTITE 350, Henkel Japan Ltd.). Finally, an acryl plate attached to the QCR force sensor, strain body, and the artificial retinal model (Mitsui Chemicals, Inc.) were integrated to produce the eye module (Fig. 8e).

As a result, the electrodes of the QCR were packaged with cover wafers of quartz crystals. In addition, the QCR force sensor itself was packaged in the eye module between the strain body and the acryl plate, as shown in Fig. 3a. This packaging method led to a fluctuation range of the sensor output comparable to 1.11 Hz or 0.22 mN (Fig. 3c); therefore, it is thought packaging had little influence on the stability of the sensor. Furthermore, its stability over 3 min also indicates that the residual stress on the sensor derived from the fabrication process was small.

#### Oscillation circuit

Figure 8f shows a schematic of the oscillation circuit driving the QCR force sensor. To oscillate a QCR continuously to avoid attenuation, a Colpitts oscillation circuit that generates oscillation and amplifies the signal is often utilized. In addition, we used a push-pull form, high-pass filter, and LC band path filter to suppress the harmonic distortion of the signal and obtain a high Q-factor.

The push-pull formed circuit took a symmetrical composition. The Colpitts oscillation circuit domain comprised two tank capacitors with $C_1$ = 27 pF and $C_2$ = 4 pF, an emitter resistor with $R_{E1}$ = 6.2 kΩ, resistors for current feedback bias with $R_1$ = 33 kΩ and $R_2$ = 68 kΩ, and an NPN bipolar transistor 1 (2SC5662, ROHM CO., Ltd.). The high-pass filter had elements with $C_{H1}$ = 5 pF and $R_{H1}$ = 100 kΩ. The LC bandpass filter had elements with resistances of $R_{B1}$ = 100 ${{\Omega }}$ and *R*_*B2*_ = 100 Ω, an inductance of *L*_*B*_ = 0.56 µH, and a capacitance of $C_B$ = 9 pF.

### Experimental device setup

The frequency of the voltage output of the QCR force sensor was read using a frequency counter (53220A, Keysight Technologies Inc.) (Figs. 4a and 5a). The QCR force sensor was calibrated against a load cell (LTS-50GA, Kyowa Electronic Instruments Co., Ltd.) attached to a micromanipulator (Quick Pro, Micro Support Co., Ltd.) (Fig. 4a). In the ILM peeling demonstration, video images of the operation obtained through the ophthalmological microscope were recorded using a USB camera (Basler ace acA 1920-150uc, Basler AG) (Fig. 5a).

### Optimization of compensation system parameters

To evaluate the validity of using the compensation method in contact detection, we recorded all instances of contact by the forceps, based on observation of the force signal and video information, as a reference. In all, we recorded a total of 40 contacts. If a detected contact moment in state 0 was earlier than the reference contact moment, detection was assumed to be accurate; if the detected contact moment was later than the reference, the detection was regarded as a failure. The number of referential moments that were not detected and the extra detected moments around which there were no referential moments were also counted. By sweeping all combinations of $F_{thr}$ from 3 to 15 mN in steps of 1 mN, values of $b_{thr}$ from 0.5 to 6 mN/s in steps of 0.5 mN/s, values of $R_{thr}^2$ from 0.05 to 0.6 in steps of 0.05, values of $\Delta t_1$ from 50 to 400 ms in steps of 50 ms, and values of $\Delta t_1$ from 50 to 400 ms in steps of 50 ms, we were able to tune each parameter to minimize the number of inaccurate, undetected, and extraneously detected results. Based on this, the optimized parameter combination was determined to be $F_{thr}$ = 7 mN, $b_{thr}$ = 1.5 mN/s, $R_{thr}^2$ = 0.1, $\Delta t_1$ = 400 ms, and $\Delta t_2$ = 200 ms. To calculate the offset value of the signal, $\Delta t_{offset}$ was defined as 100 ms.

## Supplementary information

Supplementary information

Supplementary Movie 1

Supplementary Movie 2
