# Supplementary material for: A force measurement platform for a vitreoretinal surgical simulator using an artificial eye module integrated with a quartz crystal resonator
Source: Microsyst Nanoeng. 2022 Jul 5;8:74. doi: 10.1038/s41378-022-00417-8 (PMC9256705; doi:10.1038/s41378-022-00417-8)
Supplement: Supplementary file 1 — Supplementary information [file 41378_2022_417_MOESM1_ESM.docx]

**Supplementary Information**

**Force Measurement Platform for Vitreoretinal Surgical Simulator Using Artificial Eye Module Integrated with Quartz Crystal Resonator**

*Yuta Taniguchi^1*^, Hirotaka Sugiura^1^, Toshiro Yamanaka^1^, Shiro Watanabe^1^, Seiji Omata^2^,*

*Kanako Harada^3^, Mamoru Mitsuishi^1^, Tomoyasu Shiraya^4^, Koichiro Sugimoto^4^, Takashi Ueta^4^,*

*Kiyohito Totsuka^4^, Fumiyuki Araki^4^, Muneyuki Takao^4^, Makoto Aihara^4^ and Fumihito Arai^1^*

^1^Department of Mechanical Engineering, The University of Tokyo, 7-3-1 Hongo, Bunkyo-ku, Tokyo, 113-8656, Japan

^2^Faculty of Advanced Science and Technology, Kumamoto University, 2-39-1 Kurokami, Chuo-ku, Kumamoto-shi, Kumamoto, 860-8555, Japan

^3^Center for Disease Biology and Integrative Medicine, The University of Tokyo, 7-3-1 Hongo, Bunkyo-ku, Tokyo, 113-0033, Japan.

^4^Department of Ophthalmology, The University of Tokyo, 7-3-1 Hongo, Bunkyo-ku, Tokyo, 113-8655, Japan.

*Email: taniguchi-yuta3801@g.ecc.u-tokyo.ac.jp

**Supplementary Movies:**

Movies show the videos of the bottom of the eye module embedded on Bionic-EyE using ophthalmological microscope and contact force signals obtained from the QCR force sensor during ILM peeling simulation by the untrained participant.

**Supplementary Movie 1**: The force signal and video of the motion producing a flap of the ILM.

**Supplementary Movie 2**: The force signal and video of the motion peeling the ILM by grasping a produced flap.
